# Validation of the Vitamin B_6_ Profile (Pyridoxine, Pyridoxal, and Pyridoxamine) Analysis in Rice Consumed in Korea: Effects of Cooking and Variety

**DOI:** 10.3390/foods14030457

**Published:** 2025-01-31

**Authors:** Minyoung Je, Hui Jin Lee, Jiyeon Chun

**Affiliations:** 1 Department of Food Science and Technology, Sunchon National University, Suncheon 57922, Republic of Korea; alsdud456789@naver.com (M.J.); hij16mm@naver.com (H.J.L.); 2 Bio-Healthcare Food Research and Analysis Center, Sunchon National University, Suncheon 57922, Republic of Korea; 3 Glocal University Project Team, Sunchon National University, Suncheon 57922, Republic of Korea

**Keywords:** rice, retention, vitamin B_6_ profile, variety, method validation

## Abstract

Rice, a widely consumed grain, contains various forms of vitamin B_6_, including pyridoxine (PN), pyridoxal (PL), and pyridoxamine (PM). However, limited information exists on their content and distribution in rice. This study analyzed the vitamin B_6_ profile and retention of different rice varieties consumed in Korea, using sonication-assisted acid extraction and HPLC-FLD. Retention rates were calculated based on B_6_ content before and after cooking. Five rice varieties were selected: Baromi2 (brown rice), Annammi, Chucheong (white rice), New breed white Baromi2, and New breed brown Baromi2. Vitamin B_6_ content ranged from 142.92 μg/100 g (Baromi2) to 35.89 μg/100 g (Chucheong). After cooking, Annammi showed the highest retention (109.4%), with pyridoxamine retaining 116.4%. Baromi2 had the lowest retention (34.5%), with pyridoxal at 22.1%. The analytical method demonstrated excellent accuracy (recovery rate 100.0–103.4%), precision (RSDr < 3.0%, RSD_R_ < 6.7%), and linearity (R^2^ > 0.9998). The detection limits and quantification limits for the vitamin B_6_ vitamers ranged from 0.040 to 0.070 μg/100 g and 0.103 to 0.187 μg/100 g, respectively. The results showed significant variations in vitamin B_6_ profiles and individual retention rates across rice varieties, highlighting the need for more comprehensive data on B_6_ levels. Analyzing the pyridoxal, pyridoxamine, and pyridoxine provides a more complete understanding of the B_6_ profile of rice, enhancing nutritional evaluations and precision nutrition.

## 1. Introduction

Vitamins are essential for human growth and life maintenance. Since they are not synthesized by the human body, or only in minimal amounts, they must be obtained through food [1]. Vitamin B_6_, one of the water-soluble vitamins, is a coenzyme involved in the metabolism of about 100 types of amino acids, and plays an important role mainly in decarboxylase, aminotransferase, and racemization enzymes. It also performs various physiological functions, such as regulating the immune system, and in neurotransmitter synthesis and steroid hormone action. Symptoms of vitamin B_6_ deficiency include dermatitis, stomatitis, depression, kidney stones, anemia, etc., and in severe cases, neurological disorders such as mental convulsions may occur [2]. The recommended daily intake of vitamin B_6_ for Koreans is set at 0.60–0.70 mg for infants, 0.90–1.50 mg for adolescent males, 1.50 mg for adult males, 0.90–1.40 mg for adolescent females, and 1.40 mg for adult females [3]. It has also been reported that when protein requirements increase, vitamin B_6_ requirements also increase [4].

Vitamin B_6_ exists in foods as pyridoxine (PN), pyridoxal (PL), and pyridoxamine (PM), and is also found in their respective phosphorylated forms (PNP, PLP, and PMP). Among these, pyridoxine is the most abundant form. According to [5], the body absorption rates of PN, PL, and PM at pH 4 were 67%, 38%, and 36%, respectively, indicating that absorption rate varies depending on the vitamer form of vitamin B_6_. In order to accurately evaluate the level of vitamin B_6_ intake and body absorption through food, data on the content of each vitamin B_6_ vitamer must be established in advance. Vitamin B_6_ is ingested and absorbed in various derivative forms, and most of it is transported to the liver, converted to pyridoxal 5′-phosphate (PLP), and then metabolized to 4-pyridoxal acid and excreted [6]. PL and PM are heat-stable under acidic conditions, but are heat-unstable under alkaline conditions, and vitamin B_6_ rapidly decomposes when exposed to light [7]. PN, PL, and PM are structurally similar, but PN is a relatively easy form to analyze in terms of analytical technology. PN is chemically stable, does not change into various forms, and undergoes few changes during storage and processing. On the other hand, PL and PM can be converted into active forms or undergo rapid changes, which may cause variability during analysis [7,8]. For this reason, pyridoxine was selected as the main target component in food analysis, and PL and PM were excluded from the analysis. Currently, the vitamin B_6_ content analyzed in the Korea food database is based solely on PN content, and intake levels are evaluated accordingly. As a result, the Korean food nutrition composition table does not provide accurate information on total vitamin B_6_ content. The absence of such data makes it difficult to accurately assess the intake level, which limits the use of data information in the precision nutrition service industry.

Rice is one of the three major grains widely consumed worldwide, along with wheat and corn. It primarily provides energy and contains various functional phytochemicals, making it the subject for various on-going research studies [9]. Recently, as consumer interest in health has increased, the consumption of rice varieties with enhanced functionality, such as giant germ rice and colored rice, has increased [10,11]. Rice is a food that Koreans consume frequently every day, so accurate assessment of vitamin B_6_ intake levels is very important. However, accurate nutritional information on vitamin B_6_ in rice varieties consumed in large quantities in Korea is limited.

To address this issue, the present study conducted a comprehensive analysis of the vitamin B_6_ vitamers (PN, PL, PM) in rice, focusing on the accurate content determination and distribution. Changes in vitamin B6 vitamer levels after cooking were also examined. Rigorous quality control measures were implemented to ensure the reliability and accuracy of the analytical data.

## 2. Materials and Methods

### 2.1. Reagents and Sample Preparation

The standards used for the vitamin B_6_ analysis were pyridoxine hydrochloride, pyridoxal hydrochloride, and pyridoxamine dihydrochloride, all purchased from Sigma-Aldrich Co. (St. Louis, MO, USA), with a purity of over 98%. To verify the accuracy of the analytical method, the standard reference material (SRM) used was SRM 3290 (dry cat food), which was obtained from the National Institute of Standards and Technology (NIST, Gaithersburg, MD, USA). The certified reference material (CRM) used was BCR- 487 (pig liver), which was obtained from the Institute for Reference Materials and Measurements (IRMM, Retieseweg, Geel, Belgium). The quality control (QC) sample used for precision verification was a commercial infant formula (Premium with Mom, Pasteur, Seoul, Republic of Korea). All other reagents and solvents used were of GR grade or higher, and HPLC grade.

The samples included rice varieties commonly consumed by Koreans, as well as rice flour varieties, selected as part of the National Standard Food Composition Database development for Korean Dietary Patterns, established by the Rural Development Administration (RDA, Jeonju, Republic of Korea). A total of 10 samples, including raw and cooked rice from five rice varieties—Japonica (white) Cheongcheong, Koshihikari (Japonica, white), Indica (white) Annanmi, New breed white Baromi2, and New breed brown Baromi2—were used. These samples were provided by the RDA and stored at −70 °C before analysis.

The extraction of vitamin B_6_ was performed following the method of Islam et al. [12]. Briefly, 5 g aliquot of homogenized sample was weighed into a flask, and 20 mL of 10 mM ammonium formate (0.1% formic acid) solution was added. The mixture was then extracted using an ultrasonic extractor (8893-DHT, Cole-Parmer, Chicago, IL, USA) at 40 °C for 30 min. After cooling the extract, it was centrifuged at 252× *g* (5000 rpm) for 15 min at 0 °C (MF-550, Hanil Science Industrial Co., Gwangju, Republic of Korea), and the supernatant was collected into a 50 mL volumetric flask. The same extraction procedure was repeated on the residue, and the supernatant was combined in the 50 mL volumetric flask. The final volume was adjusted to 50 mL with 10 mM ammonium formate (0.1% formic acid) solution and used as the sample for HPLC analysis.

### 2.2. Vitamin B_6_ Analysis by HPLC-FLD

The analysis of vitamin B_6_ was conducted following the method of Islam et al. [12]. A 1.6 mL aliquot of the extract was placed into an e-tube and centrifuged for 10 min (SUPR-30K, Hanil Science Industrial Co.). The supernatant was then filtered through a 0.45 μm syringe filter (cellulose acetate, DISMIC-13CP, Adventec, Osaka, Japan). The filtered sample was analyzed using high-performance liquid chromatography (HPLC) equipped with a fluorescence detector (FLD, Shimadzu, Kyoto, Japan). The column used was an Imtakt Scherzo SW-C18 (150 × 4.6 mm, 3 μm, Shiseido, Kyoto, Japan), and the detection wavelengths were set at Ex_λ_ = 290 nm and Em_λ_ = 396 nm. The gradient conditions for the column mobile phases for component separation are listed in Table 1. The flow rate of the mobile phase during analysis was set to 0.7 mL/min, the injection volume was 20 μL, and the column temperature was maintained at 35 °C.

### 2.3. Method Validation

Method validation was performed according to the single-laboratory AOAC method validation guidelines [13]. The validation procedures are as follows: standard solutions were prepared at six different concentrations (0.006, 0.013, 0.025, 0.050, 0.100, and 0.200 μg/mL) and analyzed using HPLC to construct a calibration curve for quantification.

#### 2.3.1. Linearity

Linearity was assessed by repeating the analysis three times for each concentration of the diluted standard solution, and a calibration curve was created by plotting the peak area against the concentration of the standard solution. The correlation coefficient (R^2^) of the calibration curve was evaluated.

#### 2.3.2. The Limit of Detection (LOD) and Limit of Quantification (LOQ)

The limit of detection (LOD) and limit of quantification (LOQ) were determined by analyzing blank samples and calculating the signal-to-noise (S/N) ratio average and standard deviation from the HPLC chromatogram. LOD was calculated as the average S/N multiplied by three standard deviations, and LOQ was calculated as the average S/N multiplied by ten standard deviations. Values below the LOD were reported as Not Detected (ND).

#### 2.3.3. Accuracy

Accuracy was verified by comparing the reference values of vitamin B_6_ in SRM 3290 with the analytical values, and the recovery rate (%) was calculated.

#### 2.3.4. Precision

Precision was validated by independently analyzing the commercial infant formula five times per day for repeatability, and the relative standard deviation (RSDr) was used to evaluate repeatability. Reproducibility was assessed by analyzing the same sample once per day for five days and calculating the RSD_R_.

### 2.4. Analytical Quality Control

Vitamin B_6_ analysis quality control was conducted using the analysis quality control chart (QC chart), as outlined in the AOAC guidelines [13]. The QC sample (commercial infant formula) was analyzed at least 10 times to ensure that the standard deviation of the results remained within 5%. Based on the average of the 10 analysis values, upper and lower control lines (UCL and LCL) and upper and lower action lines (UAL and LAL) were established. The QC chart was maintained throughout the analysis period as an indicator for analysis quality control. The control and action lines were calculated as follows:

UCL and LCL = mean of analyte content ± 2 × SD

UAL and LAL = mean of analyte content ± 3 × SD

where SD represents the standard deviation.

### 2.5. Statistical Analysis

Statistical analysis was performed using SPSS software (Statistics Package for the Social Science, ver. 29.0 for Windows, SPSS, Inc., Chicago, IL, USA). Means and standard deviations were calculated. Significant differences between the samples were tested using one-way ANOVA at a significance level of *p* < 0.05. Post hoc analysis was conducted using Duncan’s multiple range test at a 95% confidence level.

## 3. Results and Discussions

### 3.1. HPLC-FLD Method Validation

**Figure 1:**
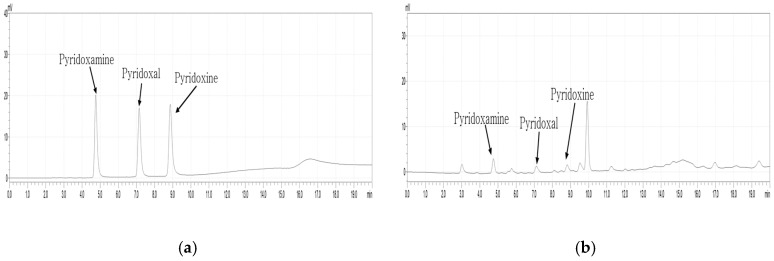
HPLC chromatograms of pyridoxine, pyridoxal, and pyridoxamine analyses. (**a**) Three standards; (**b**) rice sample (Chucheong, raw).

**Figure 2:**
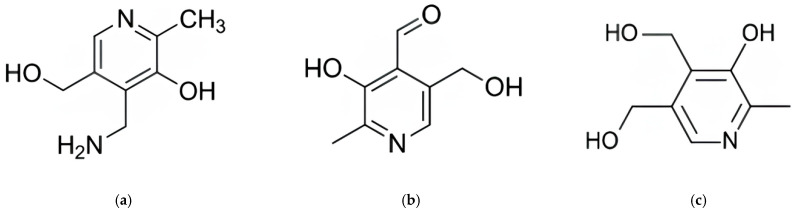
Chemical structures of (**a**) pyridoxamine; (**b**) pyridoxal; and (**c**) pyridoxine.

This study validated an HPLC-FLD method for the simultaneous analysis of three vitamin B_6_ vitamers (PN, PL, and PM) following the AOAC guidelines [13]. The method was evaluated for LOD, LOQ, linearity, accuracy, and precision. Figure 1 shows the HPLC chromatograms of vitamin B_6_ vitamers, with an elution order of PM, PL, and PN. Among the B_6_ vitamers, PM with the highest polarity was the first to be separated, appearing in the chromatogram (Figure 1a). PN, which has a weaker polarity than PM but stronger than PL, was separated second, while PN was the last to be separated. The structures of three B6 vitamers separated by HPLC are shown in Figure 2.

The results of the LOD, LOQ, and linearity verification for the three vitamin B_6_ vitamers are presented in Table 2. The LOD and LOQ values were 0.031 μg/100 g and 0.081 μg/100 g for PN, 0.042 μg/100 g and 0.102 μg/100 g for PL, and 0.002 μg/100 g and 0.053 μg/100 g for PM, respectively. All three vitamers showed excellent linearity, with correlation coefficients (R^2^) greater than 0.9998 across the analytical concentration range.

The HPLC-FLD method offers at least 15 times higher sensitivity than the HPLC-DAD method, enabling efficient analysis of trace amounts in samples [14]. Additionally, most compounds do not fluoresce, but when coupled with a fluorescent moiety, they can be analyzed with high sensitivity [15]. Vitamin B_6_ has molecular characteristics that allow it to fluoresce, making the HPLC-FLD method highly effective for detecting and quantifying trace amounts of vitamin B_6_ in food samples. In addition, Kim et al. reported LOD and LOQ values of 0.6 μg/100 g and 2.0 μg/100 g for PN, respectively, using the HPLC-FLD method [13]. Compared to this, our study method showed lower LOD and LOQ values, confirming superior sensitivity.

The accuracy of the vitamin B_6_ analysis method was evaluated using the certified reference material (SRM) 3290, which provides certified values for the three vitamin B_6_ vitamers (PN, PL, and PM) (Table 3). The PN, PL, and PM concentrations in SRM 3290 were 2754.8, 89.9, and 75.7 μg/100 g, respectively. After extraction with 10 mM ammonium formate (0.1% formic acid) and being simultaneously analyzed by HPLC-FLD, the recovery of all three vitamins ranged from 100.0% to 103.4%. According to the AOAC guidelines [13], the acceptable standard for accuracy (recovery) was 85–110%, confirming the high accuracy of the method.

The precision of the method was evaluated in terms of repeatability (RSDr) and reproducibility (RSD_R_). The RSDr and RSD_R_ values for PN, PL, and PM, as shown in Table 4, ranged from 0.7% to 4.9% and 3.0% to 3.7%, respectively. According to the AOAC guidelines [13], the acceptance criteria for accuracy (recovery) are 70–125%, with RSDr not exceeding 8%, and RSD_R_ not exceeding 16% when the analyte concentration in the sample is 1 μg per 100 g. Based on these criteria, the accuracy and precision data obtained in this study was within the acceptable range of AOAC standards.

Mann et al. reported RSDr values of 3.0% to 5.9% and RSD_R_ values of 6.7% to 11.2% for the analysis of vitamin B_6_ in soy-based infant formula using LC methods [16]. In comparison, the method applied in this study exhibited better repeatability and reproducibility. Based on the validation results, the HPLC-FLD method developed in this study can be applied to the simultaneous analysis of the three vitamin B_6_ vitamers (PN, PL, and PM) in food products containing trace amounts of vitamin B_6_, providing accurate and reproducible data.

### 3.2. Analytical Quality Control of Pyridoxine, Pyridoxal, and Pyridoxamine Analysis

**Figure 3:**
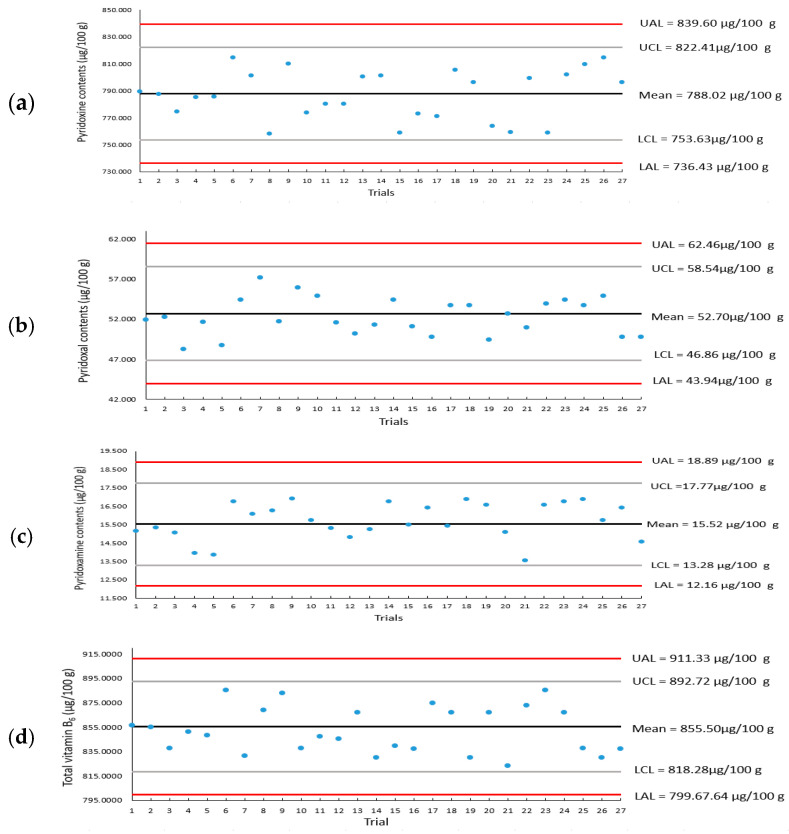
Quality control charts of HPLC-FLD for (**a**) pyridoxine; (**b**) pyridoxal; (**c**) pyridoxamine; and (**d**) total vitamin B_6_. Each blue dot represents the analysis value for that component in the quality control sample analyzed each time the sample was analyzed.

In this study, quality control (QC) charts were generated for the analysis of rice samples by including QC samples during the vitamin B_6_ analysis (Figure 3). The QC charts for PN, PL, and PM display the analytical values for each component. A cumulative QC chart for the total vitamin B_6_ analysis was constructed by summing the values of these components. All analytical values for PN, PL, and PM were within the control limits, confirming that all analyses were performed reproducibly under the validated conditions throughout the sample analysis period. 

QC charts used for quality control analysis are essential tools for ensuring and managing the quality of analytical data over the period during which the validated method is applied. They enable monitoring of results against any variables that might affect the analysis [17]. When a validated analytical method is continuously applied for repeated analysis of samples, it can be difficult to assess the accuracy and reproducibility of independent results. To address this, quality control samples with high stability and homogeneity are typically selected and analyzed alongside test samples, and a QC chart is created to monitor the data, ensuring the quality of the analytical results.

For the establishment of public databases such as national food composition databases, it is important to consider the characteristic of analyses being performed across multiple institutions over the medium-to-long term. In such studies, it is crucial that data production is sustainable over time, without being limited to a specific year or period. This ensures that data comparison and compatibility across studies are possible, and the continuous management of analytical quality is essential for maintaining the reliability of data. The vitamin B_6_ analysis values for the samples in this study demonstrate that both the method validation and analysis of quality control were rigorously conducted, indicating that the analytical data can be utilized in public databases such as the National Food and Nutrient Database.

### 3.3. Changes in Pyridoxine, Pyridoxal, and Pyridoxamine Profiles in Different Rice Varieties After Cooking

The analysis of three vitamin B_6_ vitamers (PN, PL, and PM) in different rice varieties before and after cooking, using the validated analytical method, is presented in Table 5. The contents of PN, PL, and PM in Chucheong (Japonica, white) were 7.17, 10.02, and 18.7 μg/100 g, respectively, decreasing to 1.16, 1.55, and 8.31 μg/100 g after cooking, showing the lowest B_6_ content among the five rice varieties. For Koshihikari (Japonica, white), the contents were 6.76, 9.09, and 24.17 μg/100 g, respectively, decreasing to 1.19, 1.64, and 8.43 μg/100 g after cooking. In Annammi (Indica, white), the contents were 6.38, 5.53, and 31.88 μg/100 g, respectively, decreasing to 2.23, 1.82, and 13.96 μg/100 g after cooking. For Baromi2 (New breed, white), the contents were 28.45, 14.28, and 23.35 μg/100 g, decreasing to 5.38, 1.89, and 4.88 μg/100 g after cooking. For Baromi2 (New breed, brown), the contents were 68.54, 29.99, and 44.39 μg/100 g, respectively, and decreased to 14.49, 3.67, and 9.44 μg/100 g after cooking.

The variation in vitamin B_6_ content among different plant varieties has also been observed in fruits such as mangoes, persimmons, and plums [18]. While vitamin B_6_ is essential for animals and must be obtained through the diet, plants are capable of synthesizing it autonomously. The synthesis of vitamin B_6_ in plants occurs primarily through two major pathways, resulting in the formation of pyridoxine, pyridoxal, and pyridoxamine. This process is initiated from tryptophan and proceeds through several steps, leading to the synthesis of pyridoxine and its derivatives, pyridoxal, and pyridoxamine. These pathways represent the main routes for vitamin B_6_ synthesis in plants, with enzymes involved in the formation of vitamin B_6_ derivatives playing a crucial role. Furthermore, in addition to tryptophan, other amino acids can also serve as precursors to generate various forms of vitamin B_6_, with these pathways varying across different plant species. Therefore, the final form of vitamin B_6_ can differ, depending on the species of the plant and its growth conditions.

The total vitamin B_6_ content, calculated by summing the contents of PN, PL, and PM, was highest in Baromi2 (New breed, brown) at 142.92 μg/100 g, and lowest in Chucheong (Japonica type, white) at 35.89 μg/100 g. In all samples, the contents of PN, PL, PM, and total vitamin B_6_ decreased during cooking, which can be interpreted as a result of the degradation of heat-sensitive water-soluble vitamins due to tissue breakdown and softening during heat treatment [19]. Additionally, vitamin B_6_ is known to be easily degraded under heat and alkaline conditions, which contributes to the decrease in vitamin B_6_ content after cooking [20].

**Figure 4:**
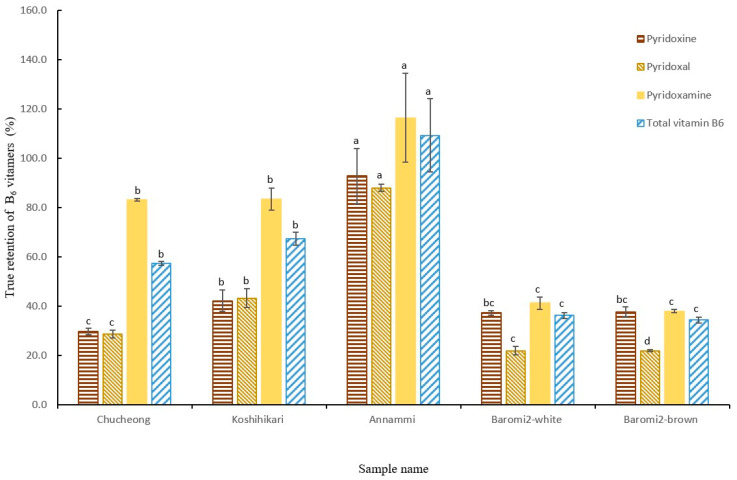
Retention of pyridoxine, pyridoxal, pyridoxamine, and total B_6_ in different varieties of rice after cooking. Means with different small letters on the same color bars for cooked rice with different varieties are significantly different at *p* < 0.05, according to Duncan’s multiple-range test (a > b > c > d > e).

The retention rates of the three B_6_ vitamers (PN, PL, PM) by rice variety after cooking are shown in Figure 4. The total vitamin B_6_ retention rate in Annammi was the highest, at 109.4%, with PM having the highest retention rate of 116.4%. In contrast, Baromi2 (New breed, brown) showed the lowest total vitamin B_6_ retention rate, at 34.5%. Chucheong, Koshihikari, and Baromi2 (white) showed retention rates of 57.5%, 67.3%, and 36.3%, respectively. Several previous studies have reported that PN generally exhibits the highest bioaccessibility, followed by PL and PM [21,22,23]. Yaman and Mızrak [5] mentioned bioaccessibility values of 76%, 53%, and 50% for PN, PL, and PM, respectively, at a gastric pH of 1.5, suggesting that bioaccessibility may vary, depending on food type. This suggests that in order to accurately assess the vitamin B_6_ intake levels in the human body, it is necessary to establish information on the content of each B_6_ vitamer in foods and consider their bioaccessibility. Furthermore, in addition to vitamin B_6_, a decrease in the biotin (vitamin B**_9_**) content in cooked noodles and cold noodles has been reported [24]. Therefore, an analysis of the actual bioavailable content of water-soluble vitamins, considering the reduction of these vitamins during cooking, is deemed necessary.

**Figure 5:**
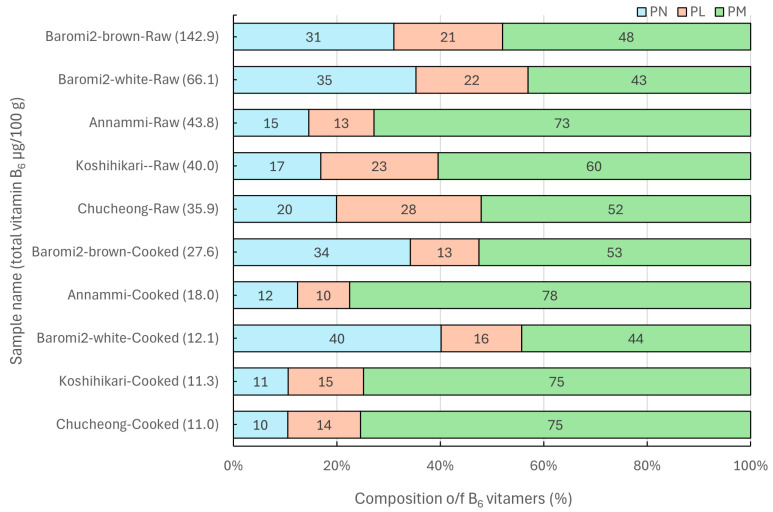
The relative composition ratio of pyridoxine (PN), pyridoxal (PL), and pyridoxamine (PM) in rice and the ranking of total vitamin B_6_ affected by cooking and variety. Total vitamin B_6_ = PN + PL + PM. Percent composition (%) of each vitamer = (the content of corresponding vitamer/total vitamin B_6_ content) × 100.

Figure 5 shows the composition ratio of total vitamin B_6_ content (PN, PL, PM) in different rice varieties, along with changes after cooking and the ranking of total content. In all rice varieties, the PM content occupied the highest proportion of the total composition in both raw and cooked rice, while the proportions of PN and PL content varied, depending on the rice variety. In the case of Baromi2 (brown), Baromi2 (white), and Annammi, the proportion of PN content was higher than that of PL, whereas in the case of Koshihikari and Chucheong, PL content was higher than PN content. According to a study by Gwak et al. [8], the analysis of the three vitamers of vitamin B_6_ in poultry and poultry products revealed that pyridoxal (PL) content was higher in most samples. This finding indicates that the levels of the three vitamers of vitamin B_6_ vary, depending on the food group. Previous analyses of vitamin B_6_ in foods have typically focused only on PN content; however, this study, which analyzed all three B_6_ vitamers in rice, revealed that PM content was, in fact, higher than PN content. This suggests that the vitamin B_6_ intake from rice has been significantly underestimated in previous evaluations. While the vitamin B_6_ profile in rice may differ, as it does in other plants and animals, many studies [25,26,27] have not provided specific information on the vitamers of vitamin B_6_**.** Therefore, it is suggested that future evaluations of vitamin B_6_ levels should involve the analysis of all three B_6_ vitamers, and a database should be established for each derivative to modify and supplement the existing data.

The recommended daily intake of vitamin B_6_ for adult men is 1.50 mg. Based on this, the intake levels calculated for a typical serving of rice (150 g) are as follows: for the rice varieties analyzed by PN content alone—Chucheong, Koshihikari, Annammi, Baromi2 (white), and Baromi2 (brown)—the intake levels were 0.116%, 0.119%, 0.223%, 0.538%, and 1.449%, respectively. When the contents of PN, PL, and PM were simultaneously analyzed, the intake levels were 1.101%, 1.126%, 1.801%, 1.214%, and 2.76%, showing a difference of 1.9 to 9.5 times. Based on the simultaneous analysis of PN, PL, and PM content in rice varieties, and considering the frequency and amount of rice consumed daily by Koreans, it is likely that the recommended daily intake of vitamin B_6_ (1.50 mg for adult men) will be adequately met [3].

Previous evaluations of vitamin B_6_ levels in food have often focused solely on PN, without considering the contents of other derivatives, leading to underestimation of the actual vitamin B_6_ content. Particularly in the case of domestic rice varieties, there has been a lack of research on vitamin B_6_ analysis. This study, by analyzing PN, PL, and PM, contributes to a more accurate evaluation of vitamin B_6_ intake.

## 4. Conclusions

This study analyzed the content and retention rate of vitamin B_6_ vitamers (pyridoxine, pyridoxal, and pyridoxamine) in cooked rice from five rice varieties commonly consumed in Korea, using HPLC with a fluorescence detector (HPLC-FLD). The HPLC-FLD demonstrated excellent accuracy (recovery rates of 100.0–103.4%), precision (RSDr < 3.0%, RSD_R_ < 6.7%), and linearity (R^2^ > 0.9998). The limits of detection (LOD) and quantification (LOQ) ranged from 0.040 to 0.070 μg/100 g and 0.103 to 0.187 μg/100 g, respectively, indicating suitability for trace vitamin analysis. Among the five rice varieties, cooked Baromi2 (brown) showed the highest vitamin B_6_ content, at 142.92 μg/100 g, while Chucheong (white) had the lowest content, at 35.89 μg/100 g. The retention rate of total vitamin B_6_ after cooking was highest in Indica rice, at 109.4%, with pyridoxamine exhibiting the highest retention among the three vitamers, at 116.4%. In contrast, Baromi2 (brown) had the lowest total vitamin B_6_ retention rate, at 34.5%, with pyridoxal showing the lowest retention rate of 22.1%. This study provided reliable data by analyzing and validating the three vitamin B_6_ vitamers in different rice varieties. The findings confirmed that pyridoxamine (PM) and pyridoxal (PL), which were previously under-represented in vitamin B_6_ content assessments, were present in significant amounts across most rice varieties. The data obtained from this analysis can be used to build a database on the missing components of vitamin B_6_.

## Figures and Tables

**Table 1:** The gradient condition of HPLC mobile phases for vitamin B_6_ analysis.

Time (min)	(A) 10 mM Ammonium Formate (0.1% Formic Acid, *v*/*v*) (%)	(B) Methanol (%)
0	100	0
14	90	10
20	90	10
21	100	0
30	100	0

**Table 2:** LOD, LOQ, and linearity of pyridoxine, pyridoxal, and pyridoxamine analyses by HPLC-FLD.

Analytes	Calibration Curve (y = ax + b) ^(1)^	Correlation of Coefficient (R^2^)	LOD ^(2)^ (µg/100 g)	LOQ ^(3)^ (µg/100 g)
Pyridoxine (PN)	y = 5,749,729 x – 10,367	0.9999	0.031	0.081
Pyridoxal (PL)	y = 4,465,178 x – 2839	1.0000	0.042	0.102
Pyridoxamine (PM)	y = 5,366,499 x – 1625	0.9999	0.002	0.053

^(1)^ y and x indicate the peak area (mAU) of the analytes in HPLC chromatograms and the concentration of corresponding analytes (mg/100 g), respectively. a: slope; b: y-intercept. ^(2)^ Limit of detection. ^(3)^ Limit of quantification.

**Table 3:** Accuracy of pyridoxine, pyridoxal, and pyridoxamine analyses using HPLC-FLD.

Sample	Analytes	Reference Value ^(3)^ (μg/100 g)	Analytical Value ^(4)^ (μg/100 g)	Recovery (%)
SRM 3290 ^(1)^	Pyridoxine (PN)	2938.0 ± 24.0	2754.8 ± 27.4	102.8
	Pyridoxal (PL)	87.7 ± 2.5	89.9 ± 0.3	102.5
	Pyridoxamine (PM)	73.7 ± 5.0	75.7 ± 2.4	100.6
CRM BCR-487 ^(2)^	Total vitamin B_6_	1930 ± 290	1730 ± 20	89.7

^(1)^ SRM 3290: standard reference material, dry cat food (NIST); ^(2)^ CRM BCR-487: certified reference material, pig liver; ^(3)^ the true value for the contents of corresponding analytes in SRM provided by NIST, IRMM; ^(4)^ the analytical values obtained in this study.

**Table 4:** Precision of pyridoxine, pyridoxal, and pyridoxamine analyses using HPLC-FLD.

Analytes	Repeatability ^(1)^		Reproducibility ^(2)^	
	Mean ± SD ^(3)^ (μg/100 g)	RSD_r_ ^(4)^ (%)	Mean ± SD (μg/100 g)	RSD_R_ ^(5)^ (%)
Pyridoxine (PN)	784.46 ± 5.79	0.7	791.57 ± 24.50	3.1
Pyridoxal (PL)	50.57 ± 1.91	3.8	54.84 ± 2.03	3.7
Pyridoxamine (PM)	14.68 ± 0.71	4.9	16.37 ± 0.49	3.0
Total vitamin B_6_	849.70 ± 7.40	0.9	861.29 ± 25.31	2.9

^(1)^ Repeatability refers to the results of five independent determinations in triplicates obtained by analyzing a QC sample five times on the same day. ^(2)^ Reproducibility refers to the results of five independent determinations in triplicates obtained by analyzing a QC sample five times on the different days (once a day). ^(3)^ SD: standard deviation. ^(4)^ RSD_r_ = 100 × (SD/mean). ^(5)^ RSD_R_ = 100 × (SD/mean).

**Table 5:** The amounts of vitamin B_6_ vitamers in different rice varieties in raw and cooked forms.

Samples	Cooking	Vitamin B_6_ Contents (μg/100 g) ^(1)^				
		Pyridoxine	Pyridoxal	Pyridoxamine	Total B_6_	PF(%) ^(2)^
Chucheong (Japonica type, white)	Raw	7.17 ± 0.16 ^c,(3)^	10.02 ± 0.20 ^c^	18.70 ± 0.11 ^e^	35.89 ± 0.23 ^e^	100.0
	Cooked	1.16 ± 0.06 ^D,(4)^	1.55 ± 0.08 ^D^	8.31 ± 0.05 ^B^	11.01 ± 0.15 ^C^	187.3
t-value ^(5)^		59.16 ***	68.67 ***	152.84 ***	153.96 ***	-
Koshihikari (Japonica, white)	Raw	6.76 ± 0.31 ^c^	9.09 ± 0.08 ^d^	24.17 ± 0.37 ^c^	40.01 ± 0.70 ^d^	100.0
	Cooked	1.19 ± 0.01 ^D^	1.64 ± 0.15 ^D^	8.43 ± 0.45 ^B^	11.26 ± 0.42 ^C^	239.6
t-value ^(5)^		29.33 ***	78.51 ***	46.91 ***	60.81 ***	-
Annammi (Indica type, white)	Raw	6.38 ± 0.03 ^c^	5.53 ± 0.18 ^f^	31.88 ± 0.14 ^b^	43.80 ± 0.18 ^c^	100.0
	Cooked	2.23 ± 0.27 ^C^	1.82 ± 0.03 ^C^	13.96 ± 2.15 ^A^	18.01 ± 2.45 ^B^	266.0
t-value ^(5)^		26.63 ***	34.48 ***	14.39 ***	18.19 ***	-
Baromi2 (New breed, white)	Raw	28.45 ± 0.48 ^b^	14.28 ± 0.35 ^b^	23.35 ± 0.54 ^d^	66.08 ± 0.87 ^b^	100.0
	Cooked	5.38 ± 0.13 ^B^	1.89 ± 0.12 ^B^	4.88 ± 0.28 ^C^	12.14 ± 0.42 ^C^	183.6
t-value ^(5)^		80.20 ***	58.16 ***	52.08 ***	96.83 ***	-
Baromi2 (New breed, brown)	Raw	68.54 ± 3.51 ^a^	29.99 ± 0.31 ^a^	44.39 ± 0.33 ^a^	142.92 ± 3.80 ^a^	100.0
	Cooked	14.49 ± 0.74 ^A^	3.67 ± 0.07 ^A^	9.44 ± 0.17 ^B^	27.60 ± 0.94 ^A^	178.4
t-value ^(5)^		26.09 ***	144.48 ***	161.03 ***	51.03 ***	-

^(1)^ Mean ± SD. ^(2)^ PF: Processing factor (%) = (weights of cooked rice/weights of raw rice)×100. ^(3)^ Means with different small letters in the same column for raw rice with different varieties are significantly different at *p* < 0.05 according to Duncan’s multiple-range test (a > b > c > d > e). ^(4)^ Means with different capital letters in the same column for cooked rice with different varieties are significantly different at *p* < 0.05 according to Duncan’s multiple-range test (A > B > C > D). ^(5)^ t-Values indicate significant differences between raw and cooked rice for the same rice variety at *** *p* < 0.001.

## Data Availability

The original contributions presented in this study are included in the article. Further inquiries can be directed to the corresponding author.

## References

[1] Kim S.H., Kim J.H., Lee H.J. (2015). Simultaneous determination of water soluble vitamins B group in health functional foods etc. by HPLC. J. Food Hyg. Saf..

[2] Coburn S.P. (1990). Location and turnover of vitamin B_6_ pools and vitamin B_6_ requirements of humans. Ann. N. Y. Acad. Sci..

[3] Ministry of Health and Welfare (MOHW), The Korean Nutrition Society (KNS) (2015). Dietary Reference Intakes for Koreans 2015.

[4] Choi S.R., Song E.J., Song Y.E., Choi M.K., Han H.A., Lee I.S., Kim H.R. (2017). Determination of Vitamin B_6_ Content using HPLC in Agricultural Products cultivated in Local Areas in Korea. Korean J. Food Nutr..

[5] Yaman M., Mızrak Ö.F. (2019). Determination and evaluation of in vitro bioaccessibility of the pyridoxal, pyridoxine, and pyridoxamine forms of vitamin B_6_ in cereal-based baby foods. Food Chem..

[6] Cho Y.O. (2010). Vitamin B_6_ requirement: Indicators and factors affecting. Korean J. Nutr..

[7] Choi S.R., Song Y.E., Han H.A., Lee S.Y., Shin S.H., Park J.J. (2019). Vitamin B_6_ content of vegetables and fruits cultivated in Korea. Korean J. Food Nutr..

[8] Gwak Y.J., Kim J., Chun J.Y. (2022). Analysis of B_6_ (pyridoxine, pyridoxal, and pyridoxamine) and B_12_ (cobalamins) vitamers in cooked chicken cuts for revision of the national food composition table. Korean J. Food Preserv..

[9] Kong S. (2009). Antioxidants in milling fractions of black rice cultivars. Food Chem..

[10] Choi I.D. (2010). Fatty Acids, Amino Acids and Thermal Properties of Specialty Rice Cultivars. J. Korean Soc. Food Sci. Nutr..

[11] Kim E.O., Oh J.H., Lee K.T., Im J.G., Kim S.S., Suh H.S., Choi S.W. (2008). Chemical compositions and antioxidant activity of the colored rice cultivars. Korean J. Food Preserv..

[12] Islam M.A., Park E., Jeong B., Gwak Y.J., Kim J., Hong W.H., Park S.J., Jung J., Yoon N.Y., Kim Y.K. (2022). Validation of vitamin B_5_ (pantothenic acid) and B_6_ (pyridoxine, pyridoxal, and pyridoxamine) analyses in seafood. J. Food Compos. Anal..

[13] AOAC (2002). AOAC Guidelines for Single Laboratory Validation of Chemical Methods for Dietary Supplements and Botanicals.

[14] Kim G.P., Hwang Y.S., Choung M.G. (2017). Quantitative analysis of vitamin B_5_ and B_6_ using high performance liquid chromatography. J. Korean Soc. Food Sci. Nutr..

[15] Harris D.C. (2010). Quantitative Chemical Analysis.

[16] Mann D.L., Ware G.M., Bonnin E., Eitenmiller R.R., Collaborators (2005). Liquid chromatographic analysis of vitamin B_6_ in reconstituted infant formula: Collaborative study. J. AOAC Int..

[17] Moon H.G., Islam M.A., Chun J. (2019). Analysis of retinol, β-carotene, vitamin E, and cholesterol contents in steamed and braised dishes of the Korean diet. Korean J. Food Preserv..

[18] Lee K.H., Gwak Y.J., Chun J.Y. (2023). Profiling of major B_6_ vitamers (Pyridoxine, Pyridoxal, and Pyridoxamine) in fruits commonly consumed in Korea. J. Korean Soc. Food Sci. Nutr..

[19] Kim G.P., Lee J.W., Ahn K.G., Hwang Y.S., Choi Y.M., Chun J.Y., Chang W.S., Choung M.G. (2014). Differential responses of B vitamins in black soybean seeds. Food Chem..

[20] Namgoong S., Kim K.D., Kim J.S. (2006). Health Functional Food.

[21] Gregory M.E. (1959). The Effect of Heat on the Vitamin B6 of Milk: I. Microbiological Tests. J. Dairy Res..

[22] Smith L.D., Grag U. (2017). Disorders of Vitamins and Cofactors. Biomarkers in Inborn Errors of Metabolism.

[23] Wozenski J.R., Leklem J.E., Miller L.T. (1980). The metabolism of small dose of vitamin B_6_ in Men 1-3. J. Nutr..

[24] Pyeon J.Y., Yu J.H., Park E.J., Choi Y.M., Kim Y. (2024). Biotin contents of grain products consumed in Korea. J. Korean Soc. Food Sci. Nutr..

[25] Jang Y.H., Jeon J.S., Lee S.H., Choi Y.M., Choung M.G. (2022). Evaluation of the vitamin B content of edible insects. J. Korean Soc. Food Sci. Nutr..

[26] Shin Y.B., Choung M.G. (2023). Determination of the pantothenic acid and pyridoxine contents in seasoning products. J. Korean Soc. Food Sci. Nutr..

[27] Jeon J.S., Jang Y.H., Choung M.G. (2023). Pantothenic acid and pyridoxine contents differ in cuts of pork. J. Korean Soc. Food Sci. Nutr..

